# Patterns and drivers of leaf carbon, nitrogen, and phosphorus stoichiometry in the desert steppe of the Ili River Basin

**DOI:** 10.3389/fpls.2026.1892050

**Published:** 2026-08-18

**Authors:** Yanxin Yang, Shiya He, Tiantian Wu, Adilaimu Abulaiti, Shanshan Xu, Junjie Tian, Xuhui Tang, Xiaofang Ye, Jie Wang, Fei Yu, Huixia Liu

**Affiliations:** 1 School of Life Sciences, Xinjiang Normal University, Urumqi, China; 2 International Center for the Collaborative Management of Cross-border Pests in Central Asia, Urumqi, China; 3 Tacheng, Research Field (Migratory Biology), Observation and Research Station of Xinjiang, Tacheng, China; 4 Urumqi Meteorological Bureau; Urumqi Pastoral Meteorological Experimental Station, Central Tianshan Grassland Ecology Monitoring Laboratory, Urumqi, Xinjiang, China

**Keywords:** desert steppe, Ili River Basin, leaf stoichiometry, spatiotemporal variation, stoichiometric homeostasis

## Abstract

**Introduction:**

Leaf carbon (C), nitrogen (N), and phosphorus (P) stoichiometry serves as a critical indicator of plant nutrient utilization strategies, underpinning grassland management and maintaining ecosystem stability in desert steppes. However, the spatiotemporal variations and multi factor driving mechanisms of leaf stoichiometry in arid desert steppes remain insufficiently explored, leaving regional adaptive mechanisms poorly understood.

**Methods:**

Here, we investigated the leaf C, N, and P stoichiometric traits of desert steppe vegetation in the Ili River Basin across May, July, and September and evaluated their spatiotemporal patterns along with their responses to hydrothermal, edaphic, and topographic factors.

**Results:**

The results showed that the mean concentrations of leaf C, N, and P were 420.93 ± 81.59 g•kg^-1^, 19.40 ± 5.94 g•kg^-1^, and 1.73 ± 0.77 g•kg^-1^, with average C/N, C/P, and N/P mass ratios of 24.92 ± 8.66, 292.13 ± 127.69, and 13.60 ± 7.44, respectively. Seasonally, leaf C continuously declined throughout the growing season, leaf N peaked in May before decreasing, and leaf P presented a unimodal trend. Spatially, leaf C was concentrated in the central and western regions; leaf N was higher in the west and lower in the east, while leaf P displayed the reverse spatial gradient. Mechanistically, leaf P response to temperature and light conditions supported the Temperature Plant Physiology Hypothesis (TPPH). Meanwhile, topography shaped regional nutrient patterns, supporting the Biogeochemical Hypothesis (BGH). Soil pH was identified as the primary driver regulating stoichiometric ratios by modulating nutrient bioavailability. Furthermore, plant competition and soil nutrient buffering jointly stabilized stoichiometric homeostasis.

**Discussion:**

This study deepens our understanding of leaf stoichiometry in the desert steppe and serves as a reference for nutrient regulation and ecological conservation in fragile arid grasslands.

## Graphical Abstract

**Figure:**
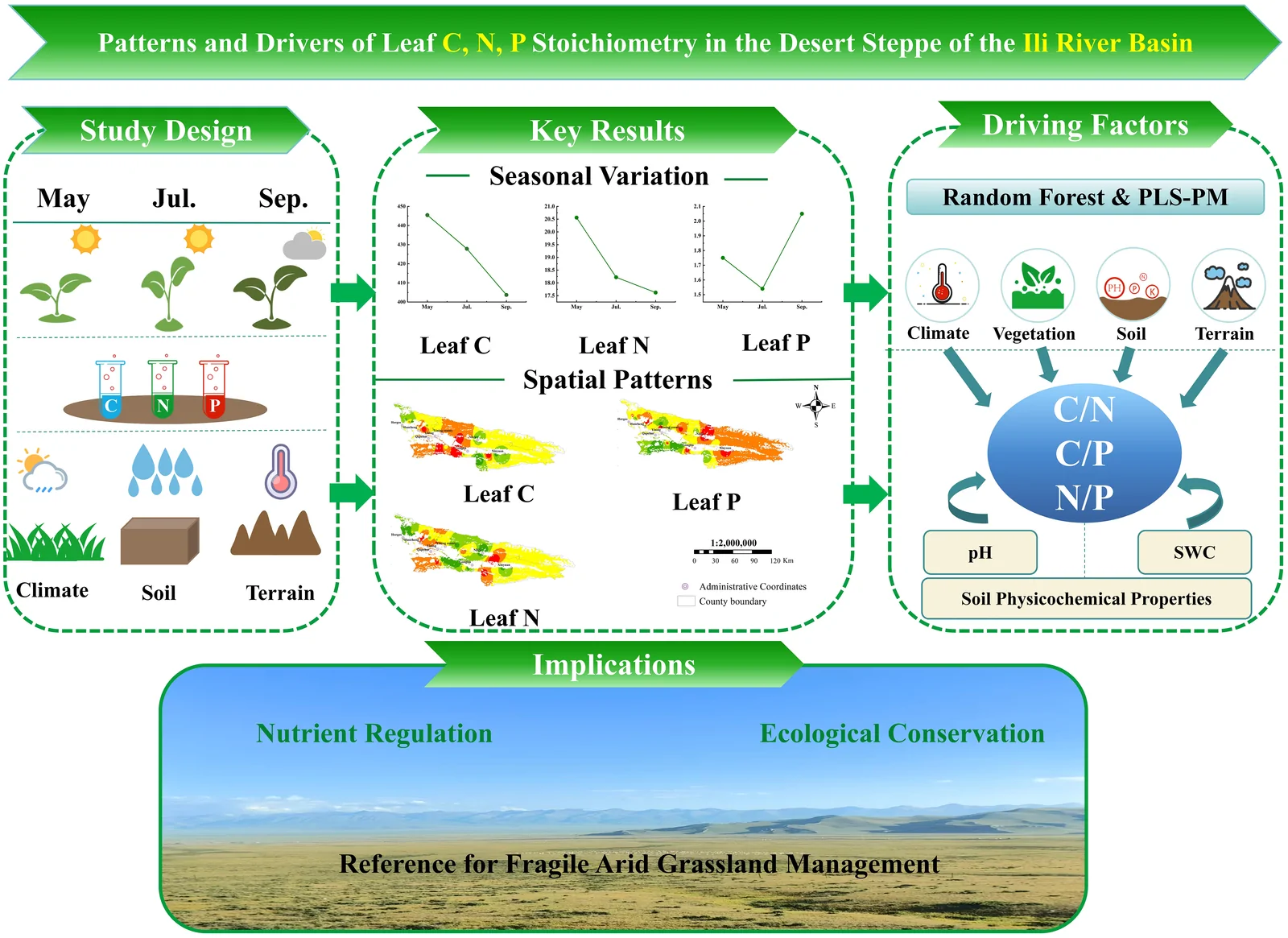

## 1 Introduction

Carbon (C), nitrogen (N), and phosphorus (P) are fundamental to terrestrial biogeochemical cycles, regulating plant growth strategies, nutrient limitations, and ecosystem functions (Wang et al., 2022; Chen et al., 2024). Leaf C:N:P stoichiometric characteristics serve as crucial biological indicators for understanding vegetation responses and adaptation to environmental fluctuations under global climate change (Peng et al., 2022; Tian et al., 2024a). Clarifying the spatiotemporal patterns and driving mechanisms of leaf stoichiometry relies heavily on established theoretical hypotheses, which provide a foundation for predicting ecosystem dynamics in response to environmental change.

Classic theoretical hypotheses—including the Biogeochemical Hypothesis (BGH), the Temperature-Plant Physiology Hypothesis (TPPH), and the Growth Rate Hypothesis (GRH)—offer distinct interpretations of spatial variations of leaf C:N: P stoichiometry and systematically reveal their regulatory mechanisms. Briefly, the BGH attributes geographic shifts in leaf stoichiometry to soil nutrient availability shaped by regional bedrock weathering and large-scale biogeochemical cycling processes (Woods et al., 2003; Reich and Oleksyn, 2004; Brantley et al., 2011; Ågren et al., 2012); the TPPH explains leaf nutrient shifts via temperature-modulated photosynthetic physiology, where cold environments induce higher leaf N concentrations to sustain metabolic efficiency (Reich and Oleksyn, 2004). Meanwhile, the GRH links leaf C:P and N:P ratios to plant growth rate and phosphorus allocation for ribosomal protein synthesis (Elser et al., 2000, 2010). Collectively, this regulatory framework has been continuously refined and validated by global syntheses and recent empirical studies (Güsewell, 2004; Isanta‐Navarro et al., 2022; Meng et al., 2025). Nevertheless, these single-driver frameworks face limitations in extremely arid and semi-arid regions. First, water serves as the main limiting factor and interacts with nutrient availability and temperature regimes, undermining the single-variable premise of classical theories (Newman and Hart, 2015). Severe drought inhibits soil mineral weathering and plant nutrient acquisition, thereby questioning the universal validity of the biogeochemical hypothesis (BGH) by decoupling soil nutrient cycling from plant elemental demand across long-term aridity gradients (Delgado-Baquerizo et al., 2013). Under prolonged water deficit, shrubs prioritize carbon allocation to drought-resistant secondary metabolites over photosynthetic nitrogen pools, weakening the generality of the stable temperature–leaf nitrogen coupling postulated by the temperature–plant physiology hypothesis (TPPH) (Yuan et al., 2022).

The spatiotemporal dynamics of plant C:N:P stoichiometry are critical for comprehending terrestrial nutrient cycling and plant environmental adaptation (Zhang et al., 2021). Nutrient distribution in China’s terrestrial ecosystems is significantly scale-dependent, with zonally based spatial heterogeneity. Among the three types of natural ecosystems—forests, shrublands and grasslands—the total nitrogen pool across all components was highest in grasslands (3,295.8 Tg N), followed by forests (2,634.9 Tg N) and lowest in shrublands (873.0 Tg N); total phosphorus stocks were also highest in grasslands (1,443.0 Tg P), followed by forests (981.1 Tg P), and shrublands had the lowest stocks (381.8 Tg P). These three ecosystem types nationwide had national total nitrogen and phosphorus stocks of 6,803.6 Tg and 2,806.0 Tg, respectively. Additionally, phosphorus density followed a similar pattern to nitrogen density, with leaf nitrogen density being lower in the north and west of China and higher in the south and east. Numerous interrelated factors are responsible for these broad-scale stoichiometric patterns. Regional nutrient variations are shaped at the macro level by topography and climate, which alter hydrothermal regimes. At the local level, the main factor influencing leaf stoichiometric characteristics is soil properties; meanwhile, biological factors, such as plant functional diversity, further regulate the C:N:P balance at the ecosystem level (Zhang et al., 2019; Chen and Chen, 2021; Liu et al., 2021). Desert grasslands, as the dominant vegetation component in arid and semi-arid regions, are inherently vulnerable and highly responsive to environmental fluctuations (Tian et al., 2024b). Vegetation in these ecosystems frequently faces dual water and nutrient limitations, and leaf C:N:P stoichiometry represents a vital adaptive strategy that mediates the trade-off between plant growth and stress defence (Cheng and Yu, 2025). By adjusting their C, N, and P allocations, desert grassland plants can prioritise growth to occupy ecological niches or improve stress tolerance to withstand harsh arid conditions. Consequently, their stoichiometric dynamics are critical for assessing regional ecosystem stability and sustainability. Although previous research has well documented macro-scale spatial patterns of plant stoichiometry across China, most studies focus on relatively stable grassland habitats. Consequently, systematic insights into the stoichiometric adaptation mechanisms of desert grasslands under combined water and nutrient stress remain limited. Furthermore, present research typically focuses just on the peak growing season, ignoring continuous stoichiometric changes between the green-up, strong growth, and senescence phenological stages. The poorly understood temporal adaptive processes that maintain plant physiological homeostasis, such as potential lag effects and compensatory nutrient regulation (Zhao, 2024), severely limit our overall understanding of the structural and functional sustainability of arid and semi-arid ecosystems.

Based on this, we selected the Ili River Basin—a typical arid and semi-arid region in Northwest China—as our study area. Through systematic field surveys and vegetation dynamics tracking conducted in May, July, and September, the study aims to: (1) clarify the baseline characteristics and seasonal dynamics of carbon, nitrogen, and phosphorus contents and their stoichiometric ratios in desert grassland leaves; (2) reveal the spatial heterogeneity patterns of these stoichiometric traits across the basin; and (3) analyze the influence of environmental factors—including meteorological variables, soil properties, and vegetation—on leaf carbon, nitrogen, and phosphorus content and stoichiometric ratios, identifying important drivers. The results will offer a scientific foundation for assessing the stability of desert grassland ecosystems and the responses of nutrient cycling to climate change.

## 2 Materials and methods

### 2.1 Study area overview

**Figure 1:**
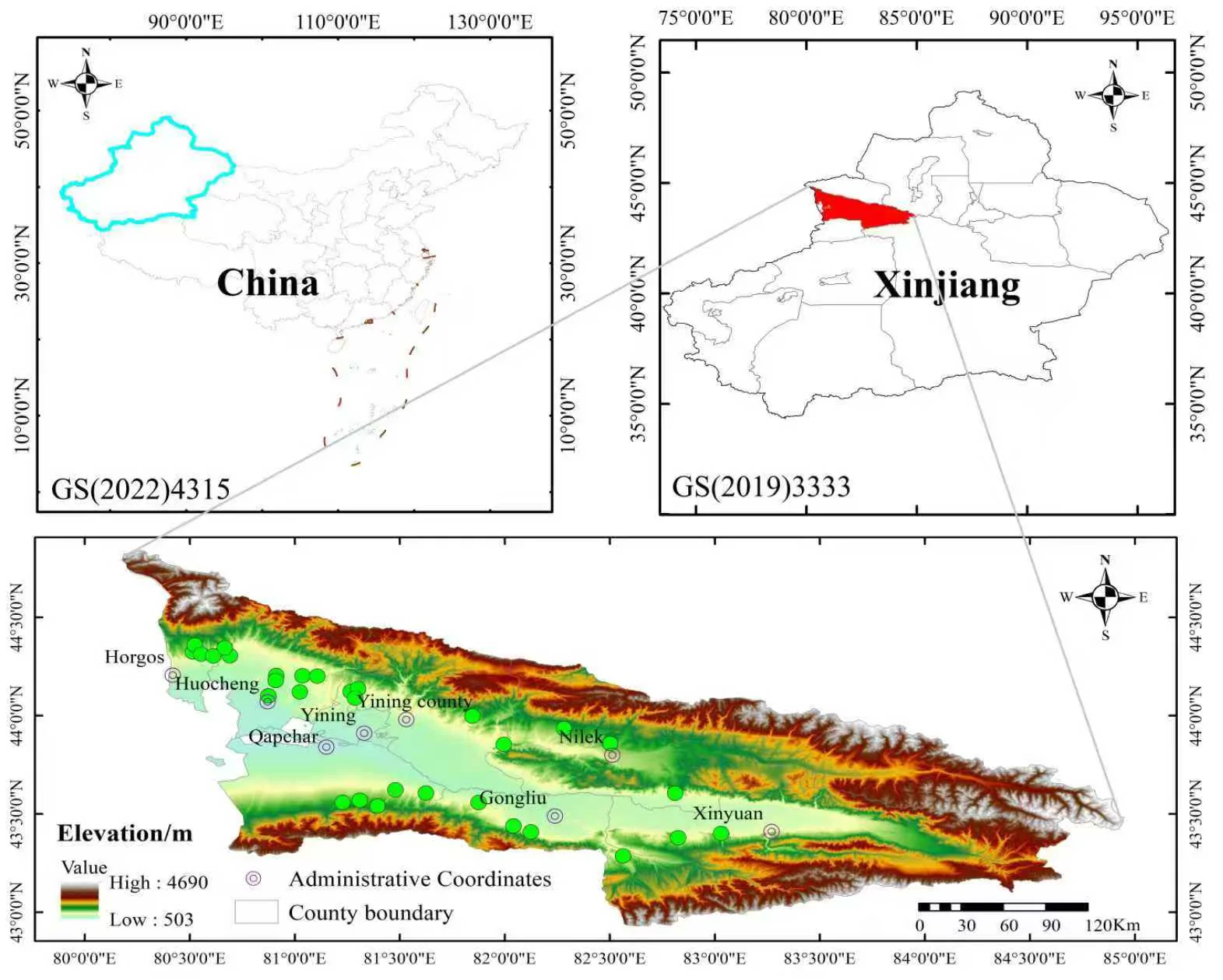
Map of sampling points in the Ili River Basin.

The Ili River Basin (42°14′16″–44°53′30″N, 80°09′42″–84°56′50″E) is located between the Bolokhoro Mountains and the Halik Mountains in the western section of the Tianshan Mountains in Xinjiang, China. The geographical location of the study area and the distribution of field sampling sites are shown in **Figure 1**. It has a temperate continental arid-semi-arid climate, with an annual average temperature ranging from 6.22°C to 9.74°C and annual precipitation between 231 mm and 504 mm. The Ili River Basin connects China and Kazakhstan and is Xinjiang’s most plentiful watershed in terms of runoff. It also serves as a bridge between steep terrain and plains, as well as desert and oasis ecosystems. Influenced by the combined effects of westerly wind moisture transport and river replenishment, it exhibits a unique “wet island” effect characteristic of arid regions. This basin is one of the world’s relatively well-preserved natural ecosystems in semi-arid regions (Liu et al., 2022; Jiang et al., 2024). Herbaceous plants and shrubs dominate the study area’s vegetation, with dominant species including *Carex miyabe*i, *Elymus nutans*, *Seriphidium transiliense*, *Sophora alopecuroides*, and *Ceratocarpus arenarius*. The grassland resources are vast, making it the major area for regional livestock development. In 2023, the livestock industry in the Ili River Valley accounted for 16.03% of Xinjiang’s total output value and 42.13% of the region’s agricultural output value (https://www.xjyl.gov.cn/xjylz), demonstrating its importance in the regional economy. In this context, vegetation carbon, nitrogen, and phosphorus levels, as well as their stoichiometric characteristics, are critical markers for evaluating pasture nutritional quality and ecological functions. These metrics are critical for understanding the mechanisms of primary productivity, identifying nutrient constraint types, and guiding the scientific management of grassland resources in the Ili River Basin.

### 2.2 Plot setup and sampling design

This study established 31 sampling sites across the desert steppe ecosystem of the Ili River Basin in 2024. Each site covered a 100 m × 100 m sampling area. Five 1 m × 1 m quadrats were deployed using a five-point sampling strategy, with one in the center and four at its diagonal corners, separated by equal intervals. All quadrats were permanent, and vegetation and soil surveys were performed at the same fixed locations during the three sampling seasons (May, July, and September). Soil sampling involved taking one topsoil sample (0–10 cm) from the center of each quadrat and homogenizing the five subsamples from each site to create a composite sample. A total of 31 composite soil samples were collected as biological replicates for each sampling season. At each site, baseline topographic and geographical characteristics (GPS coordinates, elevation, slope gradient, and aspect) were recorded.

### 2.3 Field survey and sample collection

Field surveys primarily involved vegetation community characterization and sample collection. Within each quadrat, plant height, density, canopy cover, and aboveground biomass were recorded. Plant height was measured using a tape measure combined with visual estimates. Specifically, five randomly selected, normally growing individuals of each species per quadrat were measured for their natural height, and the mean value was used to represent the average height for that species (Halbritter et al., 2024). Plant density was determined using the species-by-species counting method (Reutimann et al., 2023), while plant coverage was estimated as the percentage of the quadrat area occupied by the vertical projection of plant aboveground parts. Aboveground biomass was harvested by clipping at ground level and weighed to fresh weight (Fayiah et al., 2019). Plant and soil samples were collected concurrently for subsequent laboratory analysis. In the laboratory, soil samples were air-dried, impurities were removed, and sieving was performed. Plant samples were processed to determine leaf functional traits and chemical metrics.

### 2.4 Laboratory measurements of plant and soil samples

### 2.4.1 Plant functional traits and leaf stoichiometric traits

This study employed standardized sampling and measurement procedures to assess functional traits in 71 herbaceous plant species across 23 families and 66 genera (**Supplementary Table 1**). Seven key traits were measured: leaf thickness (LT, mm), leaf area (LA, cm²), leaf length (LL, cm), leaf width (LW, cm), leaf carbon content (LCC, g·kg^−^¹), leaf nitrogen content (LNC, g·kg^−^¹), and leaf phosphorus content (LPC, g·kg^−^¹).

During the field sampling, leaf morphological traits were measured directly to avoid changes caused by water loss. Leaf length, width, and thickness were measured using a digital vernier caliper (accuracy 0.01 mm). Each sample was measured three times, and the average value was recorded (Cornelissen et al., 2003; Schrader et al., 2021). Leaf area was measured using a non-destructive portable leaf area meter (Laser Area Meter CI-203; CID Inc.). The instrument was calibrated prior to measurement, and raw data were recorded (Suarez et al., 2025). Following field measurements, the collected plant samples were transported to the laboratory and oven-dried at 80°C for 72 hours to determine their dry weight (Hussain et al., 2019). The dried samples were pulverized using a ball mill, labeled, and stored for subsequent chemical analysis (Senawong et al., 2023).

For chemical traits, leaf carbon content was determined using the potassium dichromate external heating method (Nóbrega et al., 2015). According to the NY 525-2012 standard, a 5-fold dilution factor for the sample extracts and a standard oxidation correction factor of 1.5 were applied to the titration results to obtain the true organic carbon content. Leaf nitrogen and phosphorus contents were determined using the potassium dichromate-sulfuric acid digestion method and the perchloric acid digestion followed by the molybdenum antimony anticoloration method, respectively (He et al., 2023).

### 2.4.2 Soil physicochemical properties

Soil bulk density was determined using the cutting ring method (Blake and Hartge, 1986). Soil samples were oven-dried at 105°C to a constant weight, and soil water content (SWC) was calculated based on the mass difference before and after drying (Dettmann et al., 2021). Fresh soil samples designated for chemical analysis were air-dried naturally and cleared of impurities such as stones and plant debris. After passing through a 2 mm sieve, the samples were measured for soil pH, electrical conductivity (EC), salinity, and total dissolved solids (TDS) using standard electrometric meters. Additionally, soil particle size distribution (silt, sand, and clay contents) was determined using a laser particle size analyzer (Polakowski et al., 2023). A subsample of the sieved soil was further ground to pass a 0.149 mm (100-mesh) sieve for determination of soil organic carbon (SOC), soil total nitrogen (STN), and soil total phosphorus (STP). These parameters were quantified using the potassium permanganate oxidation method, the potassium dichromate-sulfuric acid digestion method, and the molybdenum-antimony colorimetric method, respectively (He et al., 2023; De Sousa et al., 2025).

### 2.5 Meteorological data acquisition and spatial interpolation

The meteorological data for this study were obtained from the China Meteorological Data Service Center (https://data.cma.cn/en). Data from six national meteorological stations within the Ili River Basin were selected, as their spatial distribution adequately represents the primary climatic conditions of the desert grasslands in the region. To achieve spatiotemporal alignment between the meteorological data and the vegetation/soil sampling data, ordinary Kriging was first applied to spatially interpolate the raw station data. Subsequently, interpolation residuals were corrected using inverse distance weighting (IDW). This combined approach enabled the accurate downscaling of station-level meteorological data to the specific sampling locations (Munyati and Sinthumule, 2021). The extracted meteorological variables include maximum annual temperature, minimum annual temperature, mean annual temperature, precipitation, relative humidity, wind speed, and sunshine duration. All meteorological data were aggregated on a monthly basis for subsequent analyses.

Spatial distribution maps were generated using the Inverse Distance Weighting (IDW) interpolation method. IDW is based on the assumption that known sample points closer to the prediction location exert a greater influence on the estimated value than those that are farther away (Lu and Wong, 2008). In this approach, interpolation weights are determined by an inverse power function of distance, as shown in Equation 1:

(1) $$Z_{\left(x0\right)}=\frac{\sum_{i=1}^{n}Z\left(x_{i}\right)d_{i}^{-p}}{\sum_{i=1}^{n}d_{i}^{-p}}$$

Where *Z(x0)* is the estimated value at the target location; *Z(xi)* represents the observed value at the *i-th* sample point; di denotes the distance between the target location and the sample point *i*; and *p* is the power parameter (distance decay factor). In this study, we set *p=2* and assessed the interpolation accuracy using leave-one-out cross-validation (Zhang and Ping, 2023).

### 2.6 Diversity index calculation and statistical analysis

#### 2.6.1 Vegetation diversity indices

Vegetation diversity was characterized using Simpson’s index (D), Shannon-Wiener index (H), Pielou index, and lnS index (He et al., 2022; Sun et al., 2023), with the calculation formulas shown in Equations 2–5 as follows:

Simpson(D):

(2) $$D=1-\sum_{i=1}^{n}p_{i}^{2}$$

Shannon-Wiener(H):

(3) $$H=-\sum_{i=1}^{S}P_{i}\ln P_{i}$$

Pielou:

(4) J=H/lnS

LnS:

(5) lnS

#### 2.6.2 Statistical analysis

##### 2.6.2.1 Linear mixed-effects models and statistical analysis

All statistical analyses were performed using IBM SPSS Statistics 27.0. Given that the same sampling sites were repeatedly surveyed in May, July, and September, linear mixed-effects models (LMMs) were applied to account for the non-independence of repeated observations. The sampling month was set as the fixed effect to test for seasonal differences, while the sampling site was included as a random effect (random intercept) to control for inherent spatial heterogeneity among sites. All models were fitted using restricted maximum likelihood (REML) estimation. Pairwise comparisons of estimated marginal means with Bonferroni correction were conducted to examine significant differences between months. Statistical significance was defined at *P* < 0.05. Data are presented as mean ± standard deviation (SD). Data visualizations and spatial distribution maps were generated using Origin 2024 (OriginLab Corporation, USA), ArcGIS 10.8 (ESRI, USA), and R version 4.2.0 (R Core Team, Vienna, Austria).

##### 2.6.2.2 Variable preprocessing, Random Forest modeling, and significance testing

To evaluate the relative importance of environmental and biotic factors in driving leaf stoichiometric traits, a random forest regression analysis was conducted. All predictor variables were categorized into four groups: climate factors, terrain factors, soil physicochemical properties, and vegetation factors. Prior to modeling, all variables were Z-score standardized (mean = 0, standard deviation = 1) to eliminate the influence of differing scales. Subsequently, to avoid potential multicollinearity, variables were tested using the Variance Inflation Factor (VIF). Predictors exhibiting high collinearity (VIF ≥ 10) were step-wise removed, retaining only the independent factors that were retained for the subsequent Random Forest (RF) and Partial Least Squares Path Modeling (PLS-PM) analyses.

For model training and validation, the dataset was randomly partitioned into a training set (70% of the total samples) and an independent testing set (30% of the samples). The RF models were constructed using the random forest package in R, with the number of decision trees set to 500 (ntree = 500). Model performance and predictive accuracy were evaluated on the independent testing set using the mean squared error (MSE) and the coefficient of determination (*R*^2^), as shown in Equation 6:

*R*^2^:

(6) $$R^{2}=1-\frac{MSE}{Var_{test}}$$

Where Var_test_ represents the variance of the response variable in the testing set.

To determine the statistical significance of each predictor, permutation tests with 1,000 replicates were conducted using the rfPermute package. The importance of each predictor was measured by the percentage increase in mean squared error (% IncMSE) following the permutation of that variable. The *P*-value for each variable was calculated based on the null distribution of importance scores generated from the 1,000 permuted models, and predictors with *P* < 0.05 were identified as statistically significant. All aforementioned analyses were performed in R version 4.2.0.

##### 2.6.2.3 Partial least squares path modeling analysis

Partial least squares path modeling (PLS-PM) was performed using the plspm package (version 4.1.0) in R (version 4.2.0) to elucidate the direct and indirect relationships among terrain, climate, soil properties, vegetation characteristics, and leaf stoichiometry traits.

Six independent PLS-PM models were constructed corresponding to leaf carbon (LCC), nitrogen (LNC), and phosphorus (LPC) contents, as well as their stoichiometric ratios (C/N, C/P, and N/P). All models employed a reflective scheme (Mode A) to link the latent variables with their respective observed indicators. The statistical significance of the path coefficients and weights was assessed using bootstrap resampling with 2,000 iterations. The overall fit of each model was evaluated using the Goodness-of-Fit (GOF) index, which is calculated as the geometric mean of the average communality and the average coefficient of determination (*R*^2^) of the endogenous latent variables. GOF values of 0.10, 0.25, and 0.36 were considered to indicate low, moderate, and high model fit, respectively (Tenenhaus et al., 2005; Wetzels and Odekerken, 2009).

## 3 Results

### 3.1 Overall analysis of leaf carbon, nitrogen, and phosphorus content and stoichiometric ratios

According to **Table 1**, the mean leaf carbon content was 420.93 g·kg^−^¹ (220.15–599.27 g·kg^−^¹), with a coefficient of variation (CV) of 19.38%. The mean leaf nitrogen and phosphorus contents were 19.40 g·kg^−^¹ (10.11–34.59 g·kg^−^¹) and 1.73 g·kg^−^¹ (0.62–3.82 g·kg^−^¹), exhibiting CVs of 30.63% and 44.68%, respectively. Regarding stoichiometric ratios, the mean carbon-to-nitrogen ratio was 24.92 (10.83–56.29), the mean carbon-to-phosphorus ratio was 292.13 (85.82–594.95), and the mean nitrogen-to-phosphorus ratio was 13.60 (3.03–47.61), with their corresponding CV values of 34.77%, 43.71%, and 54.69%, respectively. Based on standard CV classifications, all indicators fell within the range of moderate variation (10% < CV < 100%), and the N/P ratio displayed the highest data dispersion.

**Table 1:** Leaf carbon, nitrogen, and phosphorus contents and stoichiometric ratios characteristics.

Component	Mean	Standard deviation	Minimum value	Maximum value	Coefficient of variation (%)
Leaf carbon content (g·kg^−1^)	420.93	81.59	220.15	599.27	19.38
Leaf nitrogen content (g·kg^−1^)	19.40	5.94	10.11	34.59	30.63
Leaf phosphorus content (g·kg^−1^)	1.73	0.77	0.62	3.82	44.68
C/N	24.92	8.66	10.83	56.29	34.77
C/P	292.13	127.69	85.82	594.95	43.71
N/P	13.60	7.44	3.03	47.61	54.69

CV refers to the coefficient of variation, which quantifies the relative dispersion of data. It is calculated as $\text{CV}=\frac{\text{Standard Deviation}}{\text{Mean}}\times 100\%$. CV ≤ 10% represents weak variation, 10% < CV < 100% represents moderate variation, and CV ≥ 100% represents strong variation.

### 3.2 Seasonal dynamics of leaf carbon, nitrogen, and phosphorus content and stoichiometric ratios

**Figure 2:**
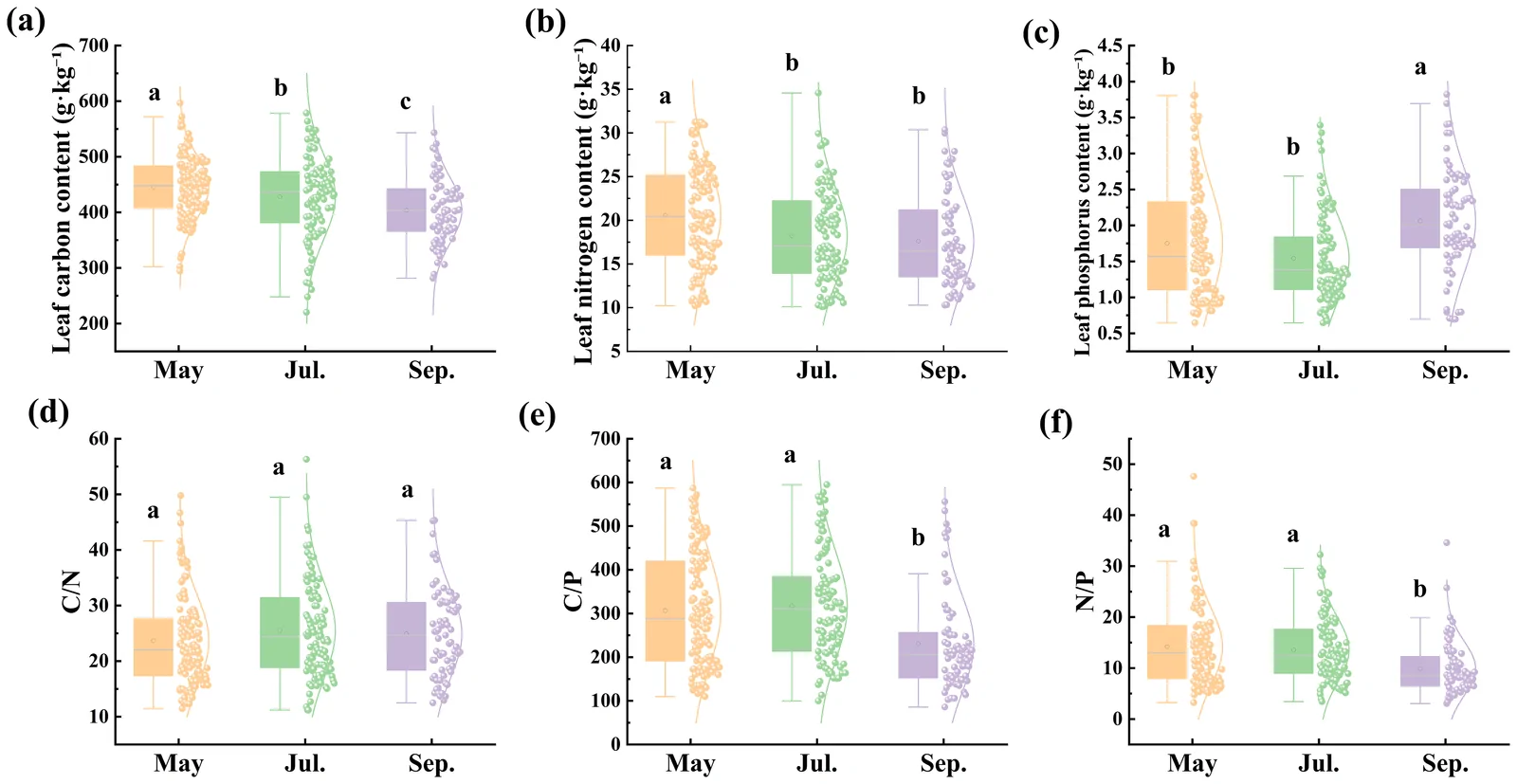
Leaf carbon, nitrogen, and phosphorus contents **(a–c)** and stoichiometric ratios **(d–f)** across different sampling months. Different lowercase letters indicate significant differences among months (*P* < 0.05).

Significant seasonal variations were observed in leaf carbon, nitrogen, and phosphorus contents (*P* < 0.05), as well as in the C/P and N/P ratios (*P* < 0.05) across May, July, and September (**Figure 2**; **Supplementary Table 2**). In contrast, leaf C/N ratio remained relatively stable across the three months, with no significant temporal variation (*P* > 0.05). Specifically, leaf carbon content peaked in May (445.46 g·kg^−^¹), being significantly higher than that in July (427.83 g·kg^−^¹) and September (403.70 g·kg^−^¹) by 4.12% and 10.35%, respectively (*P* < 0.05). Leaf nitrogen content was highest in May (20.56 g·kg^−^¹), exceeding the July (18.22 g·kg^−^¹) and September (17.62 g·kg^−^¹) values by 12.84% and 16.69%, respectively (*P* < 0.05). Conversely, leaf phosphorus content reached its maximum in September (2.05 g·kg^−^¹), showing significant increases of 17.14% and 33.12% compared to May (1.75 g·kg^−^¹) and July (1.54 g·kg^−^¹), respectively (*P* < 0.05). Regarding stoichiometric ratios, the C/P ratio showed no significant difference between May (306.45) and July (317.51), but it dropped significantly to 230.64 in September (*P* < 0.05). The leaf N/P ratio exhibited a similar downward trend over time; values in May and July did not differ significantly but were 42.66% and 36.32% higher, respectively, than those in September (*P* < 0.05).

### 3.3 Spatial distribution patterns of carbon, nitrogen, and phosphorus content and stoichiometric ratios in leaves

**Figure 3:**
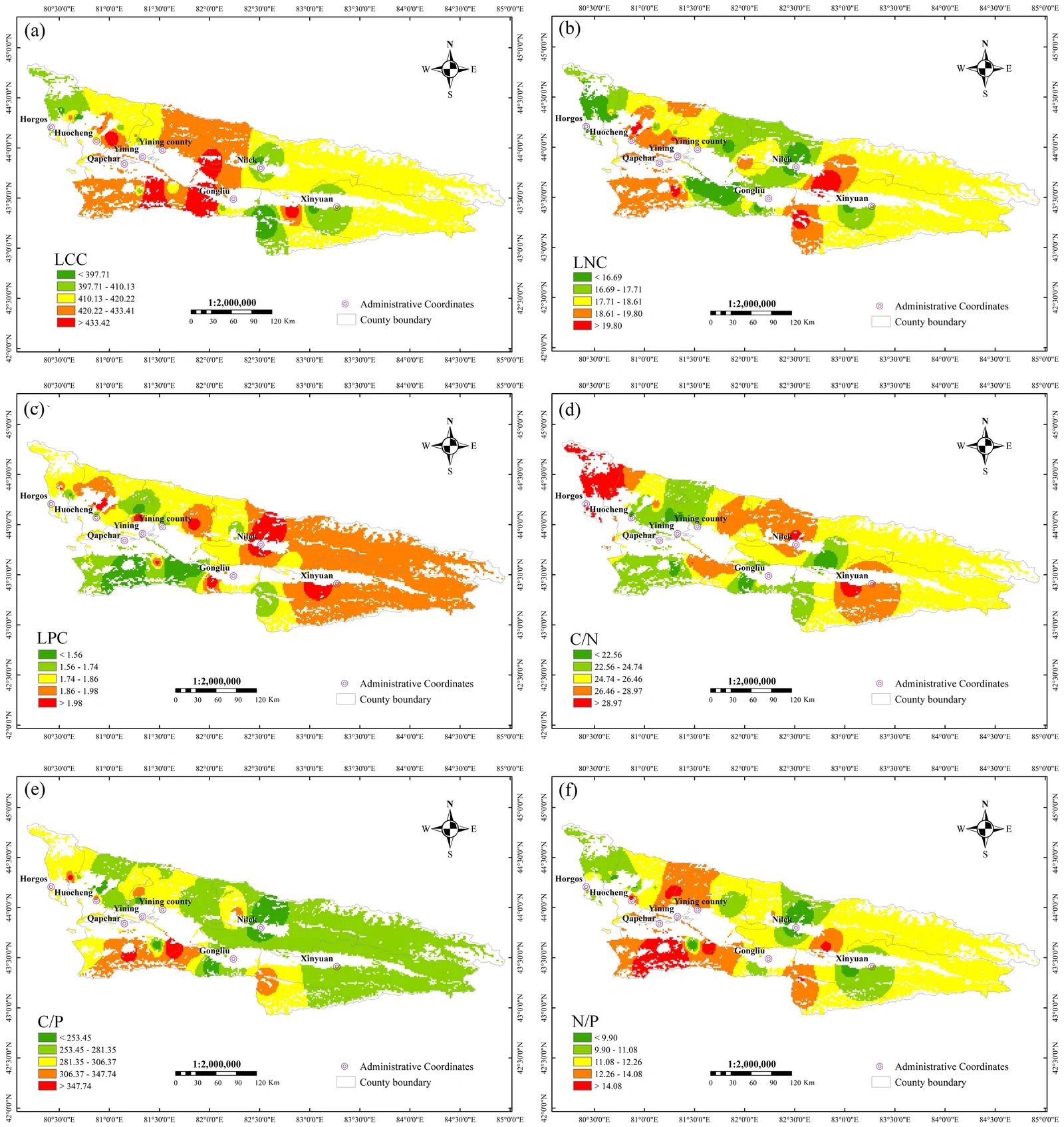
Spatial heterogeneity distribution of leaf carbon, nitrogen, and phosphorus contents and their stoichiometric ratios in the study area. The panels display the spatial variations of: **(a)** leaf carbon content (LCC, g·kg^−^¹); **(b)** leaf nitrogen content (LNC, g·kg^−^¹); **(c)** leaf phosphorus content (LPC, g·kg^−^¹); **(d)** leaf carbon-to-nitrogen ratio (C/N); **(e)** leaf carbon-to-phosphorus ratio (C/P); and **(f)** leaf nitrogen-to-phosphorus ratio (N/P).

Leaf C, N, and P contents and their stoichiometric ratios exhibited distinct spatial heterogeneity across the study area, presenting pronounced east-west zonal differentiation along the Ili River Valley. The spatial distribution pattern of leaf carbon content (LCC) showed that high-value areas were primarily concentrated in Yining County, central Gongliu County, and their adjacent regions, while low-value areas were mainly distributed in western Huocheng County, eastern Nilek County, and local areas of northern Xinyuan County (**Figure 3a**; ME = 1.34, RMSE = 11.10, Adjusted *R*^2^ = 0.79). Most regions in the eastern part of the study area were dominated by medium-level LCC. Similarly, leaf nitrogen content (LNC) exhibited high values concentrated in Xinyuan County, the Huocheng-Yining region, and their surrounding areas, whereas low-value areas were distributed across Horgos, western Huocheng, Nilek County, and northern Gongliu (**Figure 3b**; ME = −0.27, RMSE = 1.43, Adjusted *R*^2^ = 0.62). Leaf phosphorus content (LPC) displayed a continuous zonal pattern, increasing from west to east, forming a consistent east-high, west-low gradient across the valley (**Figure 3c**; ME = −0.004, RMSE = 0.11, Adjusted *R*^2^ = 0.89). Specifically, high-value LPC zones were contiguously distributed in central Nilek and the Xinyuan region, while low-value zones were located in western Huocheng and western Gongliu. Regarding the stoichiometric ratios, high-value zones for leaf carbon-to-nitrogen ratio (C/N) were primarily found in Horgos, northern Huocheng, Nilek County, Xinyuan County, and their surrounding regions, while low-value zones were concentrated in southern Qapchar, western Gongliu, and northern Xinyuan (ME = −0.18, RMSE = 2.20, Adjusted *R*^2^ = 0.75). For the leaf carbon-to-phosphorus ratio (C/P), spatial analysis revealed that high-value zones were scattered across southern Qapchar, western Gongliu, and northwestern Huocheng, with an isolated high-value patch at the southwestern margin of Xinyuan County. Conversely, low-value zones were widely distributed in eastern Yining County, Nilek, and most parts of Xinyuan (**Figure 3e**; ME = 13.45, RMSE = 42.90, Adjusted *R*^2^ = 0.47). The spatial distribution of leaf nitrogen-to-phosphorus ratio (N/P) indicated that high-value areas were concentrated in southern Qapchar and western Gongliu, with sporadic high-value patches in northwestern Yining County and western Xinyuan County, whereas low-value areas were mainly found in Horgos, eastern Yining County, Nilek, and most of southern Xinyuan (**Figure 3f**; ME = −0.39, RMSE = 1.66, Adjusted *R*^2^ = 0.64).

### 3.4 Differentiation characteristics of driving factors for carbon, nitrogen, phosphorus, and stoichiometric ratios in desert steppe vegetation of the Ili River Basin

**Figure 4:**
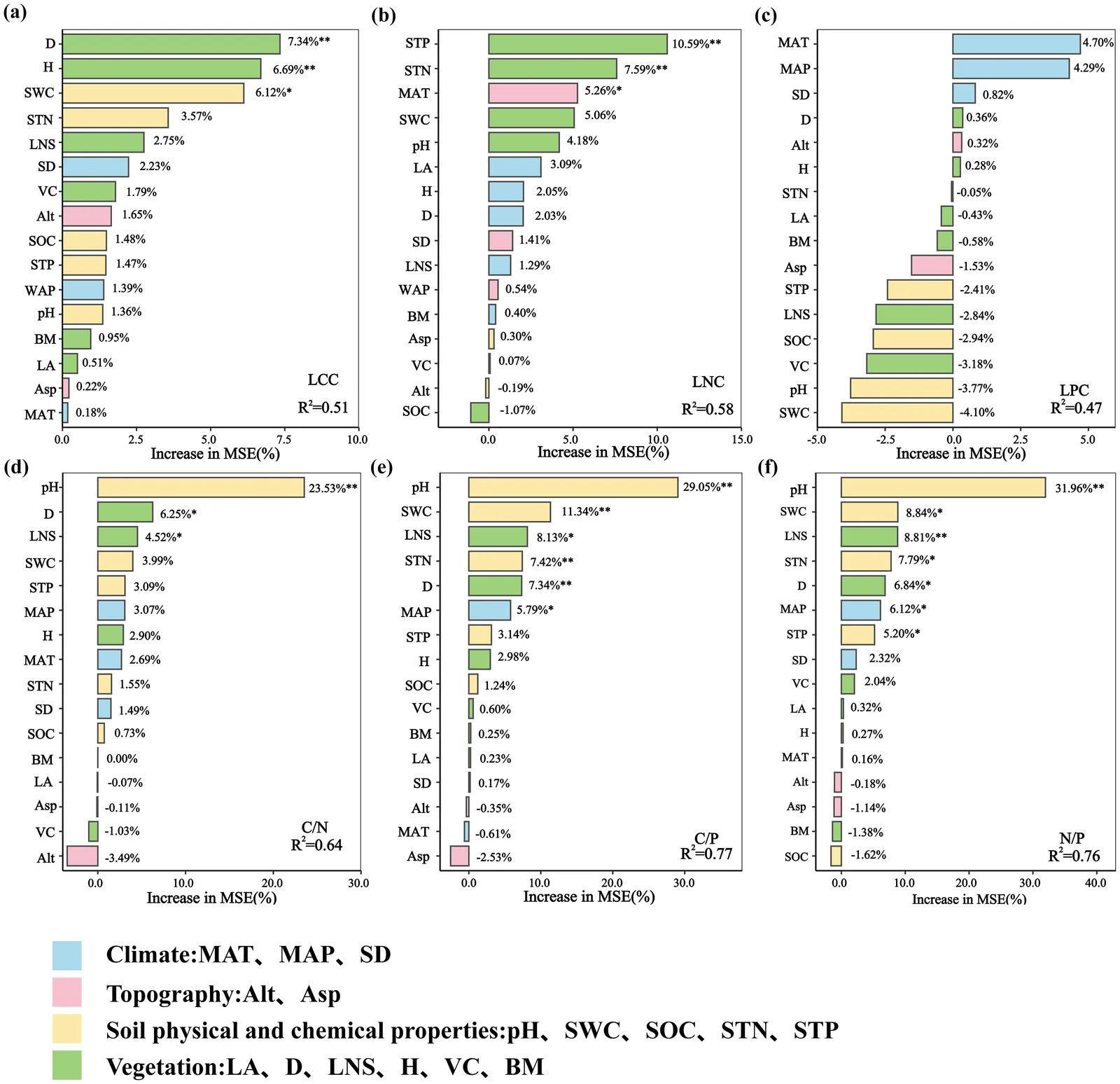
Relative importance of environmental factors in explaining leaf C, N, and P contents and their stoichiometric ratios. Random forest models were used to evaluate the effects of climate、terrain、soil physical and chemical properties, and vegetation factors on **(a)** leaf carbon content (LCC), **(b)** leaf nitrogen content (LNC), **(c)** leaf phosphorus content (LPC), **(d)** C/N, **(e)** C/P, and **(f)** N/P. The x-axis represents variable importance, expressed as the percentage increase in mean squared error (% IncMSE); higher values indicate greater contributions to model prediction. Statistical significance was determined via permutation tests (*n* = 1000 replicates): **P < 0.01, and *P < 0.05. Different colors indicate distinct categories of explanatory variables: climate, terrain, soil physicochemical, and vegetation factors. The abbreviations used in the figures are as follows: Alt, altitude; Asp, aspect; SD, sunshine duration; MAT, mean annual temperature; MAP, mean annual precipitation; D, density; LNS, lnS index; LA, leaf area; H, height; VC, vegetation coverage; BM, biomass; pH, soil pH value; SWC, soil water content; SOC, soil organic carbon content; STP, soil total phosphorus content; and STN, soil total nitrogen content.

Random Forest regression revealed the key drivers shaping leaf carbon content (LCC), leaf nitrogen content (LNC), and leaf phosphorus content (LPC) (**Figure 4**). The models explained substantial proportions of the variances in these leaf nutrient concentrations. Specifically, LCC variation was predominantly determined by vegetation traits and soil moisture, with vegetation density (D), plant height (H), and soil water content (SWC) identified as the primary drivers. In contrast, LNC was mainly controlled by soil nutrient availability and temperature, in which soil total phosphorus content (STP) and soil total nitrogen content (STN) emerged as the most critical determinants, followed by mean annual temperature (MAT). Conversely, LPC exhibited a distinct regulatory pattern, where climatic factors—particularly MAT and mean annual precipitation (MAP)—served as the leading positive predictors. Meanwhile, soil physical and chemical properties, including SWC and pH, showed negative relative importance based on the percentage increase in mean squared error (% IncMSE). For leaf stoichiometric ratios (C/N, C/P, and N/P), Random Forest models achieved higher predictive performance relative to individual leaf nutrient concentrations. Soil pH was consistently identified as the dominant driver across all three stoichiometric ratios, exerting a profound regulatory effect. Although pH overwhelmingly governed ratio variations, the secondary controlling factors differed among indices. The leaf C/N ratio was primarily modulated by vegetation characteristics, especially density (D) and the lnS index (LNS). In comparison, leaf C/P and N/P ratios were more responsive to soil moisture, precipitation, and vegetation traits, with SWC, MAP, and LNS acting as vital co-regulators.

**Figure 5:**
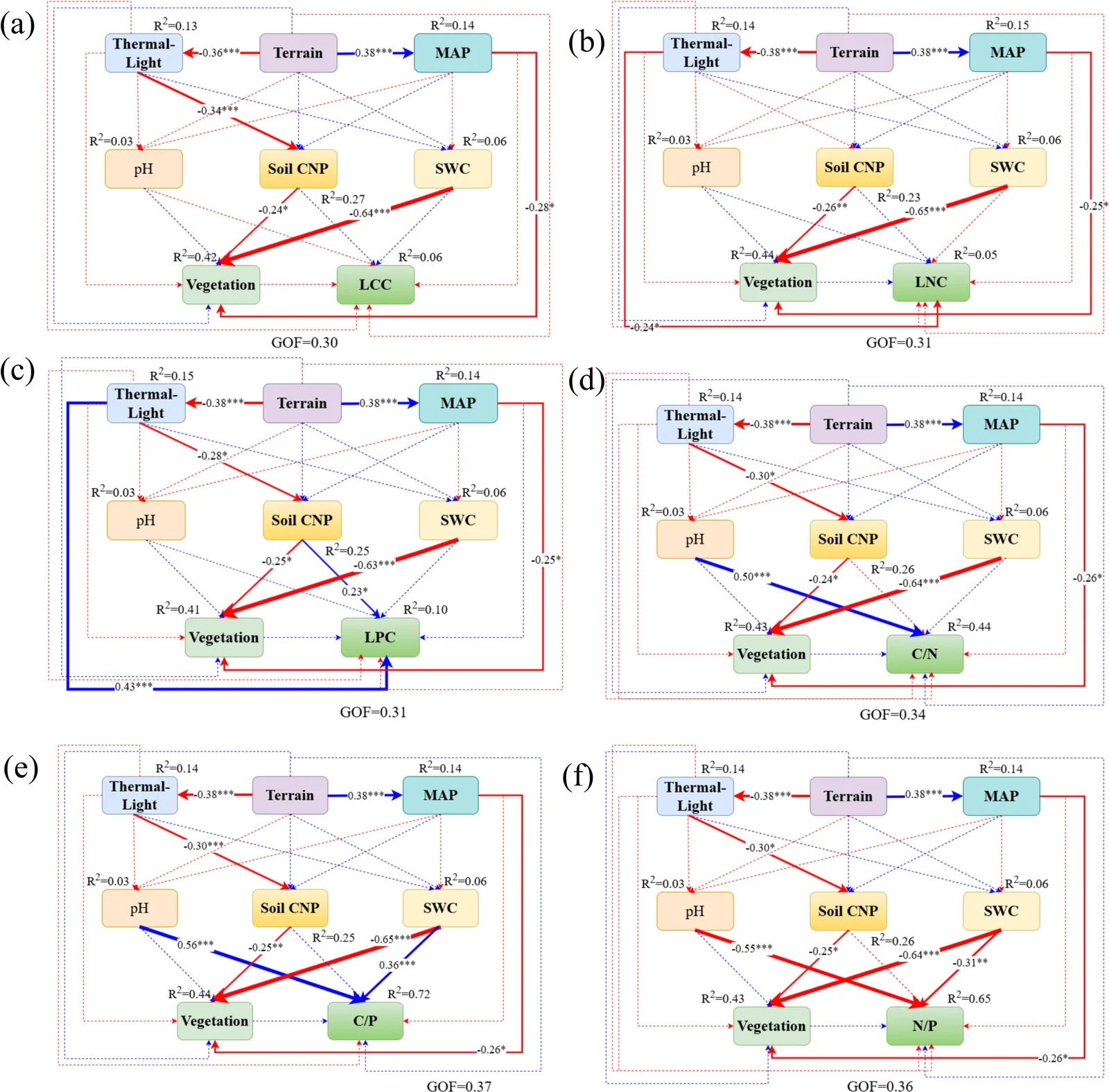
Partial least squares path modeling (PLS-PM) illustrating the direct and indirect pathways influencing leaf carbon [LCC, **(a)**], nitrogen [LNC, **(b)**], and phosphorus [LPC, **(c)**] contents and their stoichiometric ratios [C/N, **(d)** C/P, **(e)** N/P, **(f)**] (*n* = 93). Latent variables were constructed from the following observed variables: Terrain (altitude and aspect); Thermal-Light (MAT and SD); MAP; SWC; pH; Soil CNP (soil total nitrogen, phosphorus, and organic carbon contents); Vegetation (D, LNS, LA, H, VC, and BM); and the target leaf stoichiometric variables LCC, LNC, LPC, C/N, C/P, and N/P. Numbers on the arrows represent normalized path coefficients, with blue and red arrows indicating positive and negative relationships, respectively. Solid lines represent significant pathways (*P* < 0.05), and dashed lines represent non-significant pathways. *R*^2^ values represent the proportion of variance explained for each endogenous latent variable. The goodness-of-fit (GOF) value for each model is shown at the bottom of each panel. Significance levels: ***P < 0.001, **P < 0.01, *P < 0.05. Detailed path coefficients and outer model factor loadings are provided in **Supplementary Tables 4** and **S5**.

PLS-PM analysis revealed distinct driving pathways and explanatory power for leaf carbon (LCC), nitrogen (LNC), and phosphorus (LPC) contents in the Ili River Basin desert steppe, with goodness-of-fit (GOF) values of 0.30–0.31 across the three single-element models (**Figures 5a–c**). Overall, the direct impacts of local edaphic and biotic factors on individual leaf element concentrations were generally weak, resulting in low explained variances (*R*² = 0.05–0.10). Specifically, LCC was only significantly and negatively affected by soil CNP stocks (path coefficient = −0.24, *P* < 0.05), while the direct effect of vegetation characteristics was not statistically significant (path coefficient = −0.03, *P >* 0.05). For LNC, neither soil CNP nor vegetation properties exerted significant direct influences. In contrast, LPC was significantly and positively driven by vegetation characteristics (path coefficient = 0.23, *P* < 0.05) and negatively driven by soil CNP (path coefficient = −0.25, *P* < 0.05). Notably, thermal-light conditions imposed a strong direct positive effect on LPC (path coefficient = 0.43, *P* < 0.001), making leaf phosphorus the only element directly regulated by climatic factors among the three. At the regional scale, terrain consistently exerted strong indirect control: it negatively regulated thermal-light conditions (path coefficient = −0.38 to −0.36, *P* < 0.001) and positively regulated mean annual precipitation (MAP, path coefficient = 0.38, *P* < 0.001). These effects further cascaded through soil CNP, soil water content (SWC), and vegetation pathways to shape leaf elemental accumulation. SWC, in particular, acted as a critical edaphic driver of vegetation community traits, with a strong positive path coefficient of 0.63–0.65 (*P* < 0.001).

Compared with individual element contents, leaf C/N, C/P, and N/P stoichiometric ratios exhibited substantially higher explained variances (*R*² = 0.44, 0.72, and 0.65), respectively, and improved model goodness-of-fit (GOF = 0.34–0.37), indicating that environmental and vegetative drivers are more tightly coupled in regulating elemental ratios than single nutrient concentrations (**Figures 5d–f**). A core differentiating feature between individual elements and stoichiometric ratios lies in the regulatory role of soil pH. While pH showed no significant direct effect on individual leaf C, N, and P contents, it emerged as a dominant direct driver for all three stoichiometric ratios; it exerted strong positive effects on C/N (path coefficient = 0.50, *P* < 0.001) and C/P (path coefficient = 0.56, *P* < 0.001), but a strong negative effect on N/P (path coefficient = −0.55, *P* < 0.001). Vegetation characteristics also showed divergent impacts on stoichiometric traits; their effect on C/N was negligible (*P* > 0.05), whereas they strongly and positively drove C/P (path coefficient = 0.36, *P* < 0.001) and negatively drove N/P (path coefficient = −0.31, *P* < 0.01). Additionally, soil CNP consistently maintained a significant negative direct effect on all three ratios (path coefficients ranging from −0.25 to −0.24), highlighting that soil nutrient status acts as a persistent buffer restricting extreme stoichiometric shifts in desert steppe vegetation.

## 4 Discussion

### 4.1 Overall analysis of leaf carbon, nitrogen, and phosphorus content and stoichiometric ratios

This study systematically analyzed the carbon (C), nitrogen (N), and phosphorus (P) contents, as well as their stoichiometric ratios, in leaves of plants growing in the desert grasslands of the Ili River Basin. The results showed that the average leaf C, N, and P contents in this region were 420.93, 19.40, and 1.73 g·kg^−^¹, respectively. These values were generally lower than the average C, N, and P contents in Chinese terrestrial plant leaves (461.6, 20.6, and 1.99 g·kg^−^¹, respectively). However, compared to global-scale research findings (438.0, 20.2, and 1.46 g·kg^−^¹, respectively), the leaf C and N contents in our study area were marginally lower, whereas the P content was notably higher (Ma et al., 2018; Tang et al., 2018). Furthermore, compared with the desert-grassland transition zone in northern China (384.22; 23.39; 1.37 g·kg^−^¹) and the temperate desert in the Hexi Corridor (370.21; 17.88; 1.29 g·kg^−^¹) (Zhang et al., 2020; Lu et al., 2023), plant leaves in the Ili River Basin exhibited slightly higher C content, a moderate N content, and a markedly higher P content. Specifically, the leaf C content was 9.56% and 13.70% higher than in the two aforementioned regions, respectively, while the leaf P content was 26.28% and 34.11% higher. Water scarcity, a severe environmental constraint on plant productivity in the desert steppes of the Ili River Basin, induces drought stress that inhibits plant growth and development through multiple pathways. Among these, the suppression of photosynthesis is most pronounced. Drought severely impairs plant carbon metabolism by inducing stomatal closure and membrane disruption, which suppresses CO_2_ fixation and ATP synthesis while promoting photorespiratory ROS production (Farooq et al., 2009; Zahra et al., 2023). To adapt to this prolonged water limitation, plants allocate a substantial portion of their assimilated carbon to structural defence constructing highly lignified, thick-walled tissues to maintain cell turgor and minimize non-stomatal water loss. Meanwhile, drought stress disrupts the water-nutrient balance in plants, further shaping leaf N and P stoichiometry. Specifically, in the Ili River Basin, seasonal hydrological inputs from snowmelt and river seepage maintain high levels of available P (Wilson et al., 2019), providing a favorable temporal window for both carbon assimilation and soil nutrient release. This creates an environment characterized by “relative N limitation and sufficient P supply,” which directly modulates the nutrient uptake and allocation strategies of desert steppe species. Dominant plants in this region, such as *Seriphidium transiliense*, have evolved ecological strategies adapted to this arid yet seasonally replenished environment. These strategies feature moderate carbon accumulation relative to the global average, pronounced phosphorus retention, and targeted nitrogen regulation (Zhang et al., 2025b). These plants allocate sufficient C for structural water retention and lignification in their leaves, while simultaneously maintaining high leaf P pools to support physiological water regulation and metabolic turnover under pulsed water supplies. This pattern clearly demonstrates the dominant role of the desert steppe hydrothermal environment in shaping the stoichiometric traits of plant leaves (Zhang et al., 2025a).

Stoichiometric ratios, such as C/N, C/P, and N/P, can indicate plant carbon fixation efficiency and nutrient utilization strategies (Wang et al., 2024). In this study, the average C/N, C/P, and N/P ratios of desert steppe plant leaves in the Ili River Basin were 24.92, 292.13, and 13.60, respectively. Compared with the desert-grassland transition zone in northern China (C/N: 17.99, C/P: 329.85, N/P: 18.94) and the temperate desert of the Hexi Corridor (C/N: 23.37, C/P: 320.37, N/P: 14.80), the study area exhibited a slightly higher C/N ratio, a slightly lower C/P ratio, and an overall lower N/P ratio. This pattern is consistent with the slightly higher C content and markedly higher P content of plant leaves in the region. According to the growth rate hypothesis (Elser et al., 2000), the lower C/P ratio in the study area implies a relatively rapid plant growth rate. Rapid cell proliferation requires the increased consumption of N and P for synthesizing nucleic acids, proteins, and other vital substances, leading to lower C/N and C/P ratios. This stoichiometric pattern is consistent with the findings reported by (Wang et al., 2024), confirming the influence of environmental conditions on plant nutrient traits. Therefore, we hypothesize that this characteristic is associated with seasonal water replenishment in the Ili River Basin. Seasonal precipitation and meltwater improve soil nutrient availability, indirectly promoting plant growth and thus regulating leaf C:N:P stoichiometry. In this study, the mean leaf N/P ratio was 13.60, falling within the range of 10–20 proposed by (Güsewell, 2004). This indicates that the desert steppe vegetation in the Ili River Basin is co-limited by nitrogen and phosphorus, with a tendency toward nitrogen limitation. This pattern of N-P co-limitation with a nitrogen bias is intrinsically linked to the water-nutrient coupling environment shaped by seasonal water replenishment in the Ili River Basin. In accordance with the growth rate hypothesis, accelerated plant growth driven by periodic precipitation and meltwater raises nutrient demand for cell proliferation. Increased soil moisture facilitates phosphorus activation from soil minerals, while nitrogen is more prone to loss via leaching and denitrification under wet-dry cycles, causing the available nitrogen supply to lag behind growth demand (Zhao and Riaz, 2024). This nutrient limitation characteristic also reflects the adaptive strategy of desert steppe vegetation in response to seasonal hydrological fluctuations. For ecological management, targeted nitrogen supplementation can effectively alleviate nutrient constraints, providing a reference for desert steppe conservation and restoration in the basin.

### 4.2 Spatiotemporal dynamics of C, N, P and stoichiometric ratios in desert steppe plants of the Ili River Basin

The spatiotemporal dynamics of leaf C:N:P stoichiometry reveal the interplay between heterogeneous environmental stressors and plant development stages (Zhang et al., 2025c). Consistent results across arid and river-valley grasslands corroborate a conserved adaptive response in nutrient allocation. Collectively, these stoichiometric patterns underscore the critical role of regional hydrothermal conditions in governing elemental balances in terrestrial ecosystems (Sistla and Schimel, 2012). At the seasonal scale, variations in temperature and precipitation shape plant tissue stoichiometry by modulating functional metabolism throughout phenological stages (Elser et al., 2010; Tian et al., 2019). Consequently, the stoichiometry of the essential plant macronutrients carbon, nitrogen, and phosphorus serves as an effective indicator of nutrient-use status. Leaf carbon content peaked in May relative to July and September, driven by enhanced photosynthesis carbon fixation during the early growth stage. This temporal pattern links photosynthetic efficiency to phenological progression (Schurr et al., 2006; You et al., 2020). Leaf nitrogen content declined over the same early period, as rapidly proliferating leaf cells sequestered nitrogen for protein synthesis. As development proceeded, this nitrogen was progressively incorporated into accumulating biomass, reducing leaf nitrogen concentrations in mature tissues (Näsholm et al., 2009). Leaf P concentration followed a seasonal U-shaped trajectory, high in May, minimal in July, and maximal in September. Elevated May P reflected active cell proliferation and high ribosomal RNA and nucleic acid synthesis demands, aligning with the growth rate hypothesis (GRH). The July minimum arose from growth dilution caused by rapid biomass accumulation and from drought-impaired soil P mobility and root uptake. The September maximum, in turn, reflected reduced ribosome-associated P demand as development slowed, consistent with GRH predictions. Leaf C:N ratios remained stable across seasons, indicating strong homeostatic control of carbon-nitrogen allocation in desert steppe plants. By contrast, C:P and N:P ratios followed an unimodal pattern, peaking in July and declining sharply in September as leaf P rebounded. The July P minimum, diverging from typical humid-grassland patterns, reflected summer water limitation that suppressed root nutrient uptake in arid desert steppes. Nevertheless, the high N and P concentrations and low stoichiometric ratios observed during early growth corresponded to the predictions of the growth rate hypothesis.

Leaf carbon, nitrogen, and phosphorus contents and their stoichiometric ratios exhibited significant regional heterogeneity across the Ili River Basin, with both seasonal fluctuations and an east-west zonal gradient. This spatial pattern was driven primarily by the basin’s distinctive topography, which opens to the west and rises toward the east, creating a pronounced precipitation and temperature gradient in which moisture increases and temperature decreases from west to east (Li et al., 2020). Leaf phosphorus content (LPC) in this study followed a consistent east–west gradient, with higher values in the east and lower values in the west, peaking in Xinyuan and central Nilek counties. This spatial trend strongly supports the BGH and the TPPH. In the eastern regions (Nilek and Xinyuan), cooler temperatures and higher precipitation enhanced soil organic matter mineralization and phosphorus diffusion in the soil solution, thereby promoting root phosphorus uptake (He et al., 2008). In the western regions (western Huocheng), by contrast, lower LPC reflected severe drought stress. Limited soil water availability suppressed soil phosphorus mobility and root transpiration pull, ultimately constraining phosphorus transport from soil to leaves (Khan et al., 2023).

### .4.3 Influencing factors of leaf carbon, nitrogen, phosphorus, and stoichiometric ratios in desert steppe vegetation of the Ili River Basin

Our results reveal that leaf nutrient concentrations and their stoichiometric ratios in the desert steppe of the Ili River Basin are shaped by distinct driving pathways, consistent with plant physiological adaptation strategies and key ecological hypotheses. Specifically, the regulatory mechanisms underlying leaf carbon (LCC), nitrogen (LNC), and phosphorus (LPC) contents are highly divergent, exhibiting decoupled environmental responsiveness across elements. LCC was primarily constrained by plant structural traits and soil moisture, suggesting that carbon assimilation in desert steppe plants is structurally mediated and highly sensitive to local water availability under intense drought stress (Hao et al., 2026). LNC was mainly governed by soil nutrient supply and ambient temperature, consistent with the established understanding that nitrogen availability in arid and semi-arid ecosystems is tightly constrained by temperature-sensitive microbial and enzymatic mineralization of soil organic matter. This process mediates inorganic nitrogen release and ultimately shapes plant N uptake (Qiu et al., 2026). In contrast, LPC was overwhelmingly driven by thermal and light regimes. Its pronounced temperature sensitivity aligns perfectly with the temperature-plant physiological hypothesis (TPPH), which posits that plants in cold environments allocate more phosphorus to P-rich ribosomal RNA to compensate for slower biochemical reaction rates. In the warmer conditions of our study area, however, plants can maintain metabolic efficiency with a reduced biochemical phosphorus requirement, thereby allocating less P to RNA relative to functional proteins. Furthermore, rising temperatures strongly stimulate microbial phosphorus cycling in the soil; warmer conditions promote the expression of microbial functional genes, such as phoA and phoD, and the secretion of extracellular phosphatases. This accelerates organic phosphorus mobilization, ultimately enhancing plant phosphorus bioavailability in these nutrient-limited arid ecosystems (Peñuelas et al., 2026).

At the regional scale, topographic cascading effects on leaf stoichiometry align with the biogeochemical hypothesis (BGH), which states that geological and climatic templates shape plant stoichiometry by modulating soil development and weathering rates. Topographic heterogeneity serves as the core physical driver of BGH dynamics in our study area. Rather than directly modifying plant physiological processes, topography acts as a physical filter governing the spatial redistribution of solar radiation and soil water. Specifically, micro-topographic features (e.g., slope aspect and position) alter solar incidence angles to modify potential evapotranspiration while simultaneously directing surface runoff convergence and soil water infiltration (Gan et al., 2026). Steeper slopes and higher elevations experience runoff and soil erosion, restricting soil profile development to incipient A-C rather than mature A-B-C profiles and generating pronounced gradients in mineral weathering and nutrient leaching. In contrast, flat or low-lying landforms accumulate soil moisture and fine particles, promoting organic matter mineralization and deeper soil development (Luo et al., 2024). This topographically mediated redistribution of water and heat creates distinct microclimatic niches that ultimately regulate the spatial coupling between soil nutrient release and plant uptake. Collectively, the indirect topography control on leaf stoichiometry exemplifies a classic BGH cascade, where geomorphology sets the soil geochemical template, which in turn constrains or enhances plant nutrient assimilation.

Notably, leaf C/N, C/P, and N/P ratios exhibited far stronger environmental coupling than individual nutrient elements (**Figures 5d–f**), reflecting tight chemical homeostasis along environmental gradients. Across all investigated environmental and biotic factors, soil pH emerged as the dominant proximal filter governing all three stoichiometric ratios. This predominance is fundamentally tied to the dual restriction of nutrient bioavailability in the alkaline soils of the Ili River Basin. On the one hand, high soil pH promotes orthophosphate precipitation with calcium ions to form insoluble calcium phosphates, drastically reducing bioavailable phosphorus in the soil solution and inducing physiological phosphorus limitation in plants (Penn and Camberato, 2019), thereby significantly driving up leaf C/P ratios. On the other hand, elevated pH accelerates ammonia volatilization (Fenn and Kissel, 1973) and alters microbial nitrification dynamics (Nicol et al., 2008), severely reducing soil inorganic nitrogen availability and contributing to higher leaf C/N ratios. Consequently, because the pH-induced suppression of nitrogen bioavailability appears to be functionally more severe than phosphorus limitation in this ecosystem, rising soil pH ultimately results in a significant decrease in leaf N/P ratios. Overall, soil pH shapes leaf nutrient stoichiometry by concurrently modulating the chemical balances of both N and P in the soil solution.

Beyond abiotic drivers, plant community dynamics and soil nutrient stocks play crucial roles in stabilizing leaf stoichiometry. The growth rate hypothesis (GRH) posits that fast-growing plants require substantial phosphorus-rich ribosomal RNA, resulting in lower tissue C:P ratios. In this resource-limited desert-steppe, however, environmental stress profoundly constrains plant growth. As plant density and diversity rise, intensified competition for water and mobile nutrients, mediated by root proliferation and soil water depletion (Craine and Dybzinski, 2013), shifts plants to ecological strategies from rapid growth to survival and resource conservation. Consequently, plants in denser communities reduce their relative growth rates and downregulate investments in P-rich ribosomes, which perfectly explains the significant elevation of leaf C:P ratios driven by vegetation variables in our model. Meanwhile, soil C, N, and P stocks exerted consistent, strong direct negative effects on all three stoichiometric ratios. This strong negative regulation demonstrates that robust soil nutrient reservoirs act as a critical biogeochemical buffer. In nutrient-rich soils, plants avoid extreme physiological adjustments like luxury consumption or stringent nutrient conservation (Iii, 1980). Consequently, this buffering capacity minimizes tissue chemistry fluctuations, stabilizing the stoichiometric homeostasis of the desert steppe ecosystem.

## 5 Conclusion and future directions

This study elucidated the significant spatiotemporal heterogeneity of leaf C:N:P stoichiometry in the desert steppes of the Ili River Basin. In the temporal dimension, leaf carbon content continuously decreased during the growing season, leaf nitrogen content peaked in May and then declined, while leaf phosphorus content exhibited a trend of decreasing first and then increasing. The overall leaf N:P ratio (13.60 ± 7.44) fell within the range of 10–20, indicating a system co-limited by nitrogen and phosphorus, with N limitation being more pronounced. In terms of spatial patterns, high-value zones of leaf carbon content were concentrated in the central-western part of the study area; leaf nitrogen content exhibited a west-high and east-low pattern, whereas leaf phosphorus content showed an opposite spatial gradient of high in the east and low in the west.

Mechanistic analysis indicated that climatic factors, dominated by temperature and sunshine duration, were the primary drivers of spatial stoichiometric variation. Meanwhile, topography acted as the most prominent modifying factor, redistributing local hydrothermal conditions via indirect mediating pathways to shape regional nutrient variations. The study further demonstrated that biotic factors, specifically plant density and diversity, strongly regulated leaf stoichiometric ratios. In line with the growth rate hypothesis (GRH), intensified competition for resources in crowded communities compelled plants to prioritize survival over rapid growth, leading to conservative nutrient utilization strategies characterized by increased leaf C:P. Crucially, local soil nutrient stocks acted as a vital biogeochemical buffer, mediating the fluctuations in leaf stoichiometry and supporting plant nutrient homeostasis. These findings significantly advance our understanding of the biogeochemical adaptation of vegetation under water and nutrient stress in the Ili River Basin’s desert steppes, providing a robust theoretical foundation for the nutrient regulation and sustainable management of fragile arid grassland ecosystems.

However, given the high variability of community structures and the limited temporal sampling in this study, future research should incorporate long-term continuous monitoring and integrate isotopic labeling with plant functional traits to further unravel the complex coupling mechanisms shaping leaf stoichiometry in desert steppe ecosystems.

## Data Availability

The original contributions presented in the study are included in the article/**Supplementary Material**. Further inquiries can be directed to the corresponding authors.
