# Supplementary material for: Patterns and drivers of leaf carbon, nitrogen, and phosphorus stoichiometry in the desert steppe of the Ili River Basin
Source: Front Plant Sci. 2026 Aug 18;17:1892050. doi: 10.3389/fpls.2026.1892050 (PMC13529969; doi:10.3389/fpls.2026.1892050)
Supplement: Supplementary file 1 [file Table1.docx]

Supplementary Material

Patterns and drivers of leaf carbon, nitrogen, and phosphorus stoichiometry in Desert Steppe of the Ili River Basin

**Yanxin Yang 1,2,3, Shiya He 1,2,3, Tiantian Wu 1,2,3, Adilamu Abulaiti 1,2,3, Shanshan Xu 1,2,3, Junjie Tian 1,2,3, Xuhui Tang 1,2,3, Xiaofang Ye 1,2,3, Jie Wang 4, Fei Yu 1,2,3*, Huixia Liu 1,2,3***

^1^School of Life Sciences，Xinjiang Normal University，Urumqi 830054，China

^2^International Center for the Collaborative Management of Cross-border Pests in Central Asia

^3^Tacheng, Research Field (Migratory Biology), Observation and Research Station of Xinjiang, Tacheng 834700

^4^Urumqi Meteorological Bureau; Urumqi Pastoral Meteorological Experimental Station; Central Tianshan Grassland Ecology Monitoring Laboratory, Urumqi, Xinjiang 830063

*** Correspondence:**

Huixia Liu

107622023010031@xjnu.edu.cn

Fei Yu

[yufei@xjnu.edu.cn](mailto:yufei@xjnu.edu.cn)

# 1 Supplementary Figures and Tables

## 1.1 Supplementary Tables

**Table S1.** Baseline survey form of plants at key monitoring sites.

| serial number | Latin family name | Latin genus name | Latin scientific name |
| --- | --- | --- | --- |
| 1 | Asteraceae | *Seriphidium* | *Seriphidium transiliense* |
| 2 | Asteraceae | *Taraxacum* | *Taraxacum mongolicum* |
| 3 | Asteraceae | *Artemisia* | *Artemisia frigida* |
| 4 | Asteraceae | *Achillea* | *Achillea millefolium* |
| 5 | Asteraceae | *Filago* | *Filago arvensis* |
| 6 | Asteraceae | *Artemisia* | *Artemisia scoparia* |
| 7 | Asteraceae | *Chondrilla* | *Chondrilla piptocoma* |
| 8 | Asteraceae | *Artemisia* | *Artemisia gmelinii* |
| 9 | Asteraceae | *Cirsium* | *Cirsium spicatum* |
| 10 | Asteraceae | *Ligularia* | *Ligularia sibirica* |
| 11 | Asteraceae | *Aster* | *Aster hispidus* |
| 12 | Asteraceae | *Glebionis* | *Glebionis coronaria* |
| 13 | Asteraceae | *Ambrosia* | *Ambrosia artemisiifolia* |
| 14 | Poaceae | *Elymus* | *Elymus nutans* |
| 15 | Poaceae | *Bromus* | *Bromus inermis* |
| 16 | Poaceae | *Neotrinia* | *Neotrinia splendens* |
| 17 | Poaceae | *Festuca* | *Festuca ovina* |
| 18 | Poaceae | *Stipa* | *Stipa capillata* |
| 19 | Poaceae | *Poa* | *Poa annua* |
| 20 | Poaceae | *Phleum* | *Phleum pratense* |
| 21 | Poaceae | *Cynodon* | *Cynodon dactylon* |
| 22 | Poaceae | *Aegilops* | *Aegilops tauschii* |
| 23 | Poaceae | *Phragmites* | *Phragmites australis* |
| 24 | Poaceae | *Chloris* | *Chloris virgata* |
| 25 | Poaceae | *Dactylis* | *Dactylis glomerata* |
| 26 | Poaceae | *Elymus* | *Elymus sibiricus* |
| 27 | Amaranthaceae | *Ceratocarpus* | *Ceratocarpus arenarius* |
| 28 | Amaranthaceae | *Krascheninnikovia* | *Krascheninnikovia ceratoides* |
| 29 | Amaranthaceae | *Salsola* | *Salsola tragus* |
| 30 | Amaranthaceae | *Petrosimonia* | *Petrosimonia sibirica* |
| 31 | Amaranthaceae | *Grubovia* | *Grubovia dasyphylla* |
| 32 | Amaranthaceae | *Bassia* | *Bassia prostrata* |
| 33 | Amaranthaceae | *Oxybasis* | *Oxybasis glauca* |
| 34 | Fabaceae | *Trigonella* | *Trigonella arcuata* |
| 35 | Fabaceae | *Sophora* | *Sophora alopecuroides* |
| 36 | Fabaceae | *Medicago* | *Medicago sativa* |
| 37 | Fabaceae | *Alhagi* | *Alhagi camelorum* |
| 38 | Fabaceae | *Astragalus* | *Astragalus membranaceus* |
| 39 | Fabaceae | *Caragana* | *Caragana sinica* |
| 40 | Fabaceae | *Onobrychis* | *Onobrychis viciifolia* |
| 41 | Rosaceae | Potentilla | Potentilla chinensis |
| 42 | Rosaceae | Alchemilla | Alchemilla japonica |
| 43 | Rosaceae | Fragaria | Fragaria vesca |
| 44 | Brassicaceae | Strigosella | Strigosella africana |
| 45 | Brassicaceae | Chorispora | Chorispora tenella |
| 46 | Brassicaceae | Thlaspi | Thlaspi arvense |
| 47 | Lamiaceae | Thymus | Thymus mongolicus |
| 48 | Lamiaceae | *Phlomoides* | *Phlomoides umbrosa* |
| 49 | Lamiaceae | Perilla | Perilla frutescens |
| 50 | Ranunculaceae | Thalictrum | Thalictrum aquilegiifolium var. sibiricum |
| 51 | Ranunculaceae | Aconitum | Aconitum leucostomum |
| 52 | Ranunculaceae | Ranunculus | Ranunculus japonicu |
| 53 | Rubiaceae | Galium | Galium odoratum |
| 54 | Rubiaceae | Rubia | Rubia cordifolia |
| 55 | Rubiaceae | Galium | Galium spurium |
| 56 | Boraginaceae | *Lappula* | *Lappula myosotis* |
| 57 | Boraginaceae | *Myosotis* | *Myosotis alpestris* |
| 58 | Caryophyllaceae | *Arenaria* | *Arenaria serpyllifolia* |
| 59 | Caryophyllaceae | *Cerastium* | *Cerastium arvense strictum* |
| 60 | Cyperaceae | *Carex* | *Carex liparocarpos* |
| 61 | Plantaginaceae | *Plantago* | *Plantago asiatica* |
| 62 | Nitrariaceae | *Peganum* | *Peganum harmala* |
| 63 | Apiaceae | *Anethum* | *Anethum graveolens* |
| 64 | Polygonaceae | *Polygonaceae* | *Polygonum aviculare* |
| 65 | Iridaceae | *Iris* | *Iris tectorum* |
| 66 | Geraniaceae | *Geranium* | *Geranium wilfordii* |
| 67 | Convolvulaceae | *Convolvulus* | *Convolvulus arvensis* |
| 68 | Primulaceae | *Primulaceae* | *Androsace umbellata* |
| 69 | Plantaginaceae | *Veronica* | *Veronica polita* |
| 70 | Orobanchaceae | *Euphrasia* | *Euphrasia pectinata* |
| 71 | Thymelaeaceae | *Diarthron* | *Diarthron altaicum* |

**Table S2.** Leaf carbon, nitrogen, phosphorus and stoichiometric ratios (C/N, C/P, N/P) contents and coefficients of variation in different months.

| Component | Leaf carbon | | Leaf nitrogen | | Leaf phosphorus | | C/N | | C/P | | N/P | |
| --- | --- | --- | --- | --- | --- | --- | --- | --- | --- | --- | --- | --- |
|  | AV/g·kg^-1^ | CV/％ | AV/g·kg^-1^ | CV/％ | AV/g·kg^-1^ | CV/％ | AV/g·kg^-1^ | CV/％ | AV/g·kg^-1^ | CV/％ | AV/g·kg^-1^ | CV/％ |
| May | 445.46±55.56^a^ | 12.47 | 20.56±5.71^a^ | 27.80 | 1.75±0.79^b^ | 45.37 | 23.66±8.13^a^ | 34.34 | 306.45±130.56^a^ | 42.60 | 14.18±7.56^a^ | 53.33 |
| Jul. | 427.83±75.13^b^ | 17.56 | 18.22±5.39^b^ | 29.58 | 1.54±0.60^b^ | 39.05 | 25.51±8..68^a^ | 34.04 | 317.51±123.51^a^ | 38.90 | 13.55±6.32^a^ | 46.68 |
| Sep. | 403.70±57.72^c^ | 14.30 | 17.62±5.37^b^ | 30.47 | 2.05±0.73^a^ | 35.65 | 24.93±7.99^a^ | 32.07 | 230.64±109.98^b^ | 47.69 | 9.94±5.31^b^ | 53.42 |

**1.2 Supplementary Figure**

**Figure S1.** Seasonal dynamics of leaf carbon, nitrogen, and phosphorus stoichiometry in desert grasslands of the Ili River Basin. Data represent monthly averages for May, July, and September.

**Figure S2.** Spatial distribution patterns of plant leaf carbon, nitrogen, and phosphorus stoichiometry in the desert steppe of the Ili River Valley in May.

**Figure S3.** Spatial distribution patterns of plant leaf carbon, nitrogen, and phosphorus stoichiometry in the desert steppe of the Ili River Valley in July.

**Figure S4.** Spatial distribution patterns of plant leaf carbon, nitrogen, and phosphorus stoichiometry in the desert steppe of the Ili River Valley in September.

**Table S3. Mean and standard deviation of variable importance (% increase in MSE) and cross-validated R^2^ for the Random Forest models predicting leaf stoichiometry.**

| **Predictor** | **LCC(Mean ± SD)** | **LNC(Mean ± SD)** | **LPC(Mean ± SD)** | **C/N(Mean ± SD)** | **C/P(Mean ± SD)** | **N/P(Mean ± SD)** |
| --- | --- | --- | --- | --- | --- | --- |
| Alt | -0.42 ± 1.43 | 0.12 ± 1.47 | -0.38 ± 1.55 | -0.77 ± 1.33 | -0.84 ± 1.10 | -0.98 ± 1.41 |
| MAT | 1.15 ± 1.61 | 3.44 ± 1.72 | 2.91 ± 2.38 | 2.63 ± 1.94 | -0.53 ± 1.16 | -0.52 ± 1.27 |
| MAP | 0.26 ± 2.11 | 0.31 ± 1.45 | 1.95 ± 2.11 | 1.37 ± 2.11 | 2.90 ± 2.13 | 1.61 ± 1.80 |
| SD | 2.00 ± 2.65 | 2.82 ± 2.27 | 0.11 ± 1.61 | 1.25 ± 1.77 | 0.45 ± 1.52 | -0.33 ± 1.22 |
| Asp | -0.63 ± 2.13 | 0.94 ± 1.59 | -1.01 ± 1.78 | -0.47 ± 1.79 | -1.02 ± 1.45 | -0.20 ± 1.56 |
| SWC | 3.91 ± 1.32 | 4.30 ± 3.16 | -2.29 ± 1.04 | 2.61 ± 1.61 | 8.16 ± 1.66 | 5.73 ± 2.06 |
| pH | 0.87 ± 2.42 | 2.75 ± 2.57 | -1.86 ± 1.63 | 19.85 ± 2.34 | 25.40 ± 2.21 | 26.53 ± 1.68 |
| SOC | -0.53 ± 1.80 | -0.99 ± 1.58 | -1.32 ± 1.40 | 0.71 ± 1.50 | 0.41 ± 1.27 | -1.24 ± 1.34 |
| STN | 2.13 ± 2.21 | 5.06 ± 3.00 | -1.08 ± 1.66 | 1.37 ± 1.52 | 4.78 ± 1.50 | 5.60 ± 1.89 |
| STP | 0.74 ± 1.53 | 6.58 ± 4.26 | -1.01 ± 1.70 | 1.72 ± 1.85 | 3.05 ± 1.42 | 3.04 ± 1.96 |
| H | 3.99 ± 2.23 | -0.08 ± 1.70 | -1.48 ± 1.29 | 1.88 ± 1.49 | 2.15 ± 1.43 | -0.71 ± 1.13 |
| VC | 0.56 ± 1.43 | -0.56 ± 1.58 | -2.13 ± 1.24 | 0.16 ± 1.46 | 0.81 ± 1.22 | 0.98 ± 1.44 |
| D | 5.26 ± 2.95 | 0.17 ± 1.38 | -0.81 ± 1.19 | 3.92 ± 2.05 | 6.01 ± 1.69 | 4.87 ± 1.86 |
| BM | 0.68 ± 1.37 | -0.07 ± 1.04 | -1.06 ± 2.40 | -0.45 ± 1.68 | 0.94 ± 1.37 | -0.39 ± 1.25 |
| LA | 1.69 ± 2.84 | 2.05 ± 3.34 | 0.51 ± 2.06 | -0.89 ± 1.25 | 1.35 ± 2.08 | 0.32 ± 1.51 |
| LNS | 1.92 ± 1.40 | 2.14 ± 2.59 | -2.16 ± 1.28 | 5.23 ± 2.97 | 8.13 ± 1.90 | 7.76 ± 1.79 |
| Cross-validated R² | 0.45 | 0.56 | 0.40 | 0.65 | 0.76 | 0.71 |

**Note:** Values for each predictor represent the mean and standard deviation (Mean ± SD) of variable importance, measured as the percentage increase in Mean Squared Error (%IncMSE), derived from 50 repeated runs. To ensure rigorous evaluation of model stability and predictive performance, a 50-times repeated hold-out cross-validation approach (70% data for training, 30% unseen data for testing) was employed. The bottom row indicates the mean cross-validated R^2^ calculated from the test datasets across the 50 iterations.

**Table S4**. Path coefficients of major relationships and overall fit indices for the six PLS-PM models.

| **Path** | **LCC Model** | **LNC Model** | **LPC Model** | **C/N Model** | **C/P Model** | **N/P Model** |
| --- | --- | --- | --- | --- | --- | --- |
| Terrain →Thermal-Light | -0.36*** | -0.38*** | -0.38*** | -0.38*** | -0.38*** | -0.38*** |
| Terrain → MAP | 0.38*** | 0.38*** | 0.38*** | 0.38*** | 0.38*** | 0.38*** |
| Thermal-Light →Soil CNP | -0.34*** | -0.26** | -0.28* | -0.30* | -0.30*** | -0.30* |
| SWC →Vegetation | -0.64*** | -0.65*** | -0.63*** | -0.64*** | -0.65*** | -0.64*** |
| pH → Target Leaf Stoichiometry | -0.23 | 0.02 | 0.09 | 0.50*** | 0.56*** | -0.55*** |
| Soil CNP → Target Leaf Stoichiometry | -0.24* | 0.09 | -0.25* | -0.24* | -0.25** | -0.25* |
| Vegetation →Target Leaf Stoichiometry | -0.03 | 0.09 | 0.23* | -0.05 | 0.36*** | -0.31** |
| Model GOF | 0.30 | 0.31 | 0.31 | 0.34 | 0.37 | 0.36 |
| Target Variable R^2^ | 0.06 | 0.05 | 0.10 | 0.44 | 0.72 | 0.65 |

**Table S5.** Factor loadings of the observed variables across the six PLS-PM models.

| Latent Variable | Observed Variable | LCC Model | LNC Model | LPC Model | C/N Model | C/P Model | N/P Model |
| --- | --- | --- | --- | --- | --- | --- | --- |
| Terrain | Altitude | 0.944 | 0.977 | 0.964 | 0.948 | 0.961 | 0.967 |
|  | Aspect | 0.293 | 0.174 | 0.226 | 0.279 | 0.239 | 0.216 |
| Thermal-Light | MAT | 0.952 | 0.954 | 0.958 | 0.955 | 0.955 | 0.957 |
|  | SD | 0.962 | 0.960 | 0.955 | 0.959 | 0.959 | 0.957 |
| Soil CNP | SOC | 0.906 | 0.869 | 0.933 | 0.915 | 0.890 | 0.901 |
|  | STN | 0.410 | 0.415 | 0.338 | 0.406 | 0.447 | 0.433 |
|  | STP | 0.388 | 0.507 | 0.388 | 0.352 | 0.388 | 0.364 |
| Vegetation | h | 0.732 | 0.712 | 0.748 | 0.711 | 0.700 | 0.708 |
|  | D | 0.741 | 0.724 | 0.706 | 0.730 | 0.732 | 0.726 |
|  | VC | 0.831 | 0.802 | 0.816 | 0.811 | 0.806 | 0.807 |
|  | LNs | 0.628 | 0.680 | 0.653 | 0.677 | 0.686 | 0.862 |
|  | BM | 0.664 | 0.647 | 0.663 | 0.638 | 0.636 | 0.640 |

**Note:**For simplicity, single-indicator latent variables (i.e., MAP, SWC, PH, and the target leaf stoichiometry indices) are omitted from this table as their loadings and weights are all constantly 1.000.

**Table S6.** Cross-validation results of spatial interpolation for C, N, P contents and their stoichiometric ratios.

|  | ME | RMSE | Formula | R^2^ |
| --- | --- | --- | --- | --- |
| C(g/kg) | 1.344202 | 11.105544 | Predicted = 100.085139 + 0.764693*Measured | 0.79086 |
| N(g/kg) | -0.275561 | 1.434565 | Predicted = 0.242307 + 0.861955*Measured | 0.619853 |
| P(g/kg) | -0.004535 | 0.112548 | Predicted = 6.276231 + 0.634538*Measured | 0.894027 |
| C/N | -0.180005 | 2.204446 | Predicted = 6.525907 + 0.742880*Measured | 0.758626 |
| C/P | 13.455117 | 42.901721 | Predicted = 153.603566 + 0.513438*Measured | 0.4682 |
| N/P | -0.388222 | 1.668260 | Predicted = 3.780858 + 0.653841*Measured | 0.642384 |
